# Penicillin susceptibility among *Staphylococcus aureus* skin and soft tissue infections at a children’s hospital

**DOI:** 10.1128/spectrum.00869-24

**Published:** 2024-09-09

**Authors:** J. Chase McNeil, Lauren M. Sommer, Marritta Joseph, Kristina G. Hulten, Sheldon L. Kaplan

**Affiliations:** 1 Department of Pediatrics, Division of Infectious Diseases, Baylor College of Medicine and Texas Children’s Hospital, Houston, Texas, USA; Icahn School of Medicine at Mount Sinai, New York, New York, USA

**Keywords:** *S. aureus*, penicillin, skin infection, abscess, pediatric

## Abstract

Shortly after its introduction into clinical practice, *Staphylococcus aureus* isolates gained resistance to penicillin via the acquisition of β-lactamases. A number of centers have recently described an increase in the proportion of invasive methicillin-susceptible *S. aureus* (MSSA), which are also susceptible to penicillin (PSSA). Little data are available regarding the prevalence or impact of PSSA in skin and soft tissue infections (SSTI). Community-acquired MSSA SSTI isolates were obtained through a surveillance study at Texas Children’s Hospital from January 2017 to December 2021. A total of 200 random isolates underwent PCR for *blaZ* β-lactamase; *blaZ*-negative isolates then underwent penicillin susceptibility testing using macrobroth dilution. Isolates which were *blaZ* negative and had a penicillin MIC ≤0.125 µg/mL were regarded as PSSA with the remainder regarded as penicillin-resistant MSSA (PR-MSSA). All PSSA underwent multilocus sequence typing. Medical records were reviewed. The median age of subjects was 4.2 years (IQR: 1.6–10.5). PSSA accounted for 9% of isolates during the study period. PSSA and PR-MSSA cases were similar with respect to age, demographics, and rates of prior antibiotic exposure. PSSA isolates less often had vancomycin MIC ≥1.5 µg/mL. Furthermore, 39% of PSSA were variants of sequence type 1. In multivariable analyses, penicillin susceptibility was independently associated with both hospital admission and surgical intervention. PSSA account for a small but significant proportion of MSSA SSTI in children. Clinically distinguishing patients with PSSA and PR-MSSA SSTI is challenging. However, PSSA SSTI were independently associated with higher rates of hospital admission as well as the need for surgical intervention suggesting a significant clinical impact.

**IMPORTANCE:**

The vast majority of *Staphylococcus aureus* in the US are penicillin resistant with most clinical labs no longer reporting penicillin susceptibility for this organism. A number of centers, however, have reported increasing penicillin susceptibility among invasive *S. aureus* infections. Skin and soft tissue infections (SSTI) are far more common than invasive infections, yet the frequency and impact of penicillin-susceptible *S. aureus* (PSSA) in this population are uncertain. Through active surveillance at a children’s hospital, we found that 9% of methicillin-susceptible *S. aureus* SSTI isolates were PSSA. PSSA were independently associated with hospital admission for the management of SSTI as well as the need for debridement in the operating room. Given that most SSTI are managed in the outpatient setting, these findings suggest a clinical impact of this phenotype and the need for a reassessment of the value in susceptibility testing and potentially even treatment with penicillin.

## OBSERVATION

Shortly after the introduction of penicillin into clinical practice, *Staphylococcus aureus* strains developed resistance to penicillin through the acquisition of a penicillinase encoded by *blaZ* (1, 2). Currently, given the very high prevalence of penicillin resistance, most clinical laboratories in the US do not report or even perform penicillin susceptibility testing in *S. aureus* (3).

A number of centers have recently reported increasing penicillin susceptibility among invasive methicillin-susceptible *S. aureus* (MSSA) infections (4). While invasive infections are potentially life threatening, non-invasive infections account for the bulk of *S. aureus* disease in children, with skin and soft tissue infections (SSTI) outnumbering cases of invasive disease by at least 20:1 (5). We investigated the frequency and clinical significance of penicillin susceptibility in *S. aureus* SSTI at a tertiary children’s hospital.

MSSA SSTI isolates collected from January 2017 to December 2021 through an ongoing IRB-approved surveillance study at Texas Children’s Hospital (TCH) were eligible for inclusion. A waiver of informed consent was granted for this study. Methicillin susceptibility was determined using cefoxitin disk diffusion. We sought to screen at least 10% of SSTI isolates. Twenty non-duplicate community-associated (6) MSSA SSTI isolates were chosen at random from every 6-month interval during the study period.

For all isolates, PCR was performed for *blaZ*, *lukSF-PV*, and accessory gene regulator (*agr*) group (7, 8). All isolates negative for *blaZ* were subjected to penicillin broth dilution assays. Penicillin-susceptible *S. aureus* (PSSA) were regarded as MSSA isolates, which were *blaZ* negative and with penicillin MIC <0.125 µg/mL; all other isolates were regarded as penicillin-resistant MSSA (PR-MSSA) (9). All isolates underwent susceptibility testing to clindamycin, trimethoprim-sulfamethoxazole, and tetracycline using Kirby Bauer, and vancomycin and daptomycin MICs were determined using *E*-test. For PSSA, MICs to oxacillin and cefazolin were determined with broth dilution. Penicillin susceptibility testing was performed in the research context only, and results were not available to clinicians. PSSA isolates were further characterized by multilocus sequence typing.

Corresponding medical records were reviewed for all screened cases. Operative surgical intervention was regarded as any drainage/debridement procedure conducted under general anesthesia. Given that most cases of SSTI are managed as an outpatient, a major outcome of interest was infection prompting hospital admission >24 hours. A logistic regression model was used to assess risk factors for hospital admission; variables associated with hospital admission at the *P* < 0.1 level in univariable analyses were included in the regression model.

To assess the potential impact of antimicrobial selective pressure in our community, the number of outpatient antibiotic prescriptions for penicillin, amoxicillin, and ampicillin from January 2011 to December 2021 across the TCH system (including emergency departments, urgent care centers, and affiliated primary care practices) was obtained from the electronic medical record. Temporal trends in penicillin/aminopenicillin prescribing were examined using linear regression.

During the study period, 3,331 *S*. *aureus* SSTI isolates were recovered, of which 1,701 were MSSA (51.6%) with 200 undergoing penicillin susceptibility testing (200/1,701, 11.8%). Overall, 9% (*n* = 18) of tested MSSA isolates were penicillin susceptible; there was no clear temporal trend in the frequency of PSSA (Fig. S1). Among PSSA, the modal penicillin MIC was ≤0.015 µg/mL (*n* = 17/18); by contrast, the modal oxacillin and cefazolin MICs were 0.25 µg/mL (*n* = 10/18) and 1 µg/mL (*n* = 11/18), respectively. While the number of outpatient prescriptions for penicillin declined in the study period (*P* = 0.001, *R*^2^ = 0.74), prescribing of aminopenicillins increased through the end of 2019 (*P* < 0.001, *R*^2^ = 0.96) followed by a sharp decline (Fig. S2), coinciding with the onset of the SARS-CoV-2 pandemic.

The medical records of cases associated with the 200 isolates screened for penicillin susceptibility were reviewed in detail to assess clinical features and outcomes associated with PSSA. Children with PSSA and PR-MSSA SSTI were of similar demographics and had comparable rates of previous SSTI and antibiotic use and had similar body sites of infection (Table 1). Subjects with PSSA SSTI were more often admitted to the hospital (66.7% vs 41.2%, *P* = 0.04) and/or underwent operative drainage/debridement (55.5% vs 25.8%, *P* = 0.01).

**TABLE 1:** Comparison of PSSA and PR-MSSA

	All cases (*n* = 200)	PSSA (*n* = 18)	PR-MSSA (*n* = 182)	*P* value^*a*^
Median age, years (IQR)^*b*^	4.2 (1.6–10.5)	3.2 (0.8–6.2)	4.2 (1.6–10.9)	0.2
Female gender	91 (45.9)	8 (44.4)	83 (45.6)	1
Self-identified race				0.67
White	130 (65)	12 (66.7)	118 (64.8)	
African American/Black	50 (25)	4 (22.2)	46 (25.3)	
Asian	7(3.5)	0	7 (3.8)	
Any other stated race	2 (1)	0	2 (1.1)	
Unknown/not answered	11 (5.5)	2 (11.1)	9 (4.9)	
Self-identified Hispanic ethnicity	81 (40.5)	8 (44.4)	73 (40.1)	0.45
History of eczema	39 (19.5)	3 (16.7)	36 (19.8)	1
Previous SSTI	42 (21)	6 (33.3)	36 (19.8)	0.22
Antibiotics in prior 90 days	72 (36)	7 (38.9)	65 (35.7)	0.81
Specific antibiotic exposures in prior 90 days^*c*^				
Amoxicillin/ampicillin	11 (5.5)	1 (5.6)	10 (5.4)	1
Amoxicillin-clavulanate	3 (1.5)	1 (5.6)	2 (1.1)	0.25
Cephalexin	9 (4.5)	1 (5.6)	8 (4.3)	0.58
Ceftriaxone	1 (0.5)	1 (5.6)	0	0.09
Clindamycin	19 (9.5)	0	19 (10.4)	0.23
Trimethoprim-sulfamethoxazole	22 (11)	2 (11.1)	20 (10.9)	1
Macrolides	2 (1)	0	2 (1.1)	1
Vancomycin	1 (0.5)	1 (5.6)	0	0.09
Mupirocin	9 (4.5)	1 (5.6)	8 (4.3)	0.58
β-Lactam in prior 90 days	24 (12.5)	4 (22.2)	20 (10.9)	0.25
SSTI diagnosis				
Abscess	103 (51.5)	10 (55.6)	93 (51.1)	0.81
Superinfected eczema	27 (13.5)	0	27 (14.8)	0.14
Cellulitis	25 (12.5)	2 (11.1)	23 (12.6)	1
Felon/paronychia	20 (10)	1 (5.6)	19 (10.4)	1
Impetigo	16 (8)	1 (5.6)	15 (8.2)	1
Lymphadenitis	11 (5.5)	1 (5.6)	10 (5.5)	0.15
Traumatic wound infection	8 (4)	2 (11.1)	6 (3.5)	0.15
Infection prompting hospital admission	86 (43)	12 (66.7)	74 (41.2)	0.04
Length of stay, days		2 (1–3)	2 (1–3)	1
Drainage/debridement required	129 (64.5)	13 (72.2)	116 (63.7)	0.61
Operative intervention	57 (28.5)	10 (55.5)	47 (25.8)	0.01
Systemic antibiotic prescribed	192 (96)	17 (94.4)	175 (96.2)	0.54
Recurrence of SSTI in 30 days	23 (11.5)	1 (5.6)	22 (12.1)	0.7
Microbiology				
Clindamycin resistance	28 (14)	3 (16.7)	25 (13.7)	0.7
Vancomycin MIC ≥1.5 mcg/mL	143 (71.5)	9 (50)	134 (73.6)	0.05
Daptomycin MIC >1 mcg/mL	7 (3.5)	0	7 (3.8)	1
Trimethoprim sulfamethoxazole resistance	6 (3)	0	6 (3.3)	1
Tetracycline resistance	16 (8)	2 (11.1)	14 (7.7)	0.6
PVL^*d*^ positive	83 (41.5)	4 (22.2)	79 (43.4)	0.1
*agr* Group I	154 (77)	17 (94.4)	137 (75.3)	0.08
*agr* Group II	18 (9)	1 (5.6)	17 (9.3)	1
*agr* Group III	13 (6.5)	0	13 (7.1)	1
*agr* Group IV	8 (4)	0	8 (4.4)	1
*agr* Non-typeable	7 (3.5)	0	7 (3.8)	1

^*a*^ Categorical variables were compared with Fisher’s exact test and continuous variables with Wilcoxon rank sum test.

^*b*^ Categorical variables are presented as *n* (%); continuous variables are presented as median and interquartile range.

^*c*^ Antibiotic categories not mutually exclusive.

^*d*^ PVL, Panton-Valentine leukocidin.

One-hundred ninety-two subjects received treatment with systemic antimicrobials (96%). The most commonly prescribed antibiotics were clindamycin (55%), trimethoprim-sulfamethoxazole (31%), and cephalexin (19.5%); no subject received penicillin.

Eighty-six subjects (43%) required hospital admission. Subjects who were admitted to the hospital were younger (median age 2.4 vs 5.6 years, *P* = 0.002, Table 2) and more often had diagnoses of abscesses (66.3% vs 40.4%, *P* < 0.001), infection involving the neck (16.3% vs 3.5%, *P* = 0.002) or genitals (13.9% vs 2.6%, *P* = 0.005), required drainage/debridement (66.3% vs 0%, <0.001), and had infection due to PSSA (13.9% vs 5.3%, *P* = 0.04). In multivariable analyses, age <4 years (adjusted odds ration [aOR] 2.1, 95% CI 1.1–3.9), need for drainage (aOR 2.75, 95% CI 1.3–5.7), infection involving the genitals (aOR 4.7, 95% CI 1.16–18.9), and PSSA (aOR 3.1 95% CI 1.02–9.31) remained independently associated with hospital admission (Table 2).

**TABLE 2:** Associations with hospital admission for MSSA SSTI

	Hospital admission (*n* = 86)	No hospital admission (*n* = 114)	Univariable *P* value	Multivariable *P* value	aOR (95% CI)
Median age, years (IQR)	2.4 (0.9–9.9)	5.6 (2.1–10.7)	0.002		
Age <4 years^*a*^	53 (61.7)	45 (39.5)	0.003	0.02	2.1 (1.1–3.9)
Prior antibiotics	36 (41.8)	36 (31.5)	0.14		
Prior SSTI	15 (17.4)	27 (23.6)	0.3		
Diagnosis of abscess	57 (66.3)	46 (40.4)	<0.001	0.26	1.49 (0.74–3.05)
Drainage/debridement	66 (76.7)	63 (55.3)	0.002	0.007	2.75 (1.31–5.77)
Operative debridement^*b*^	57 (66.3)	0	<0.001		
Infection site					
Face	10 (11.6)	16 (14.1)	0.68		
Hand	1 (1.2)	17 (14.9)	0.47		
Neck^*b*^	14 (16.3)	4 (3.5)	0.002		
Genitals	12 (13.9)	3 (2.6)	0.005	0.03	4.7 (1.16–18.97)
PVL^*c*^ positive	36 (41.8)	37 (32.4)	0.18		
PSSA	12 (13.9)	6 (5.3)	0.04	0.04	3.1 (1.02–9.31)

^*a*^ Represented the median age for the study group.

^*b*^ Operative debridement and infection of the neck were removed from the model secondary to collinearity.

^*c*^ PVL, Panton-Valentine leukocidin.

The use of operative intervention for abscesses was examined as a secondary outcome. Operative intervention with general anesthesia was more common in younger children (age <4 years: 66.7% vs 45.4%, *P* = 0.045), those with infection involving the neck (16.7% vs 1.8%, *P* = 0.01) or genitals (28.8% vs 5.5%, *P* = 0.03), history of prior antibiotics (56.3% vs 23.6%, *P* = 0.001), and PSSA (16.7% vs 3.6%, *P* = 0.04). In a multivariable model, infection of the neck or genitals, previous antibiotic exposure, and PSSA remained significantly associated with operative intervention (Table S1).

The 18 PSSA isolates belonged to 12 different sequence types (ST), including 1 novel ST (ST8961). The most common STs were ST97 (*n* = 5), ST188 (*n* = 2), and ST8 (*n* = 2). Overall, 7/18 (38.9%) PSSA were variants of ST1.

A number of investigators have recently reported increasing *in vitro* penicillin susceptibility among MSSA infections with previous studies largely focusing on invasive disease (4, 10, 11). Observational studies conducted in Australia suggest that penicillin treatment of PSSA bloodstream infection is associated with improved outcomes relative to treatment with flucloxacillin (12), highlighting the clinical significance of this phenotype.

We previously reported that 6.7% of MSSA osteoarticular infection isolates at two pediatric centers were penicillin susceptible and that the rate of PSSA was increasing over time, reaching 20.4% by the final study year (10). A study conducted in Boston of all *S. aureus*-positive cultures found increasing rates of PSSA from 2010 to 2014, peaking at 10.4% (4). In the present study, we found that 9% of MSSA SSTI isolates were penicillin susceptible, relatively consistent with such prior work. While penicillin prescribing has declined at our center, the prescription of aminopenicillins has remained high for much of the study period, arguing against reduced β-lactam use as an explanation for rising PSSA. There was a decline in aminopenicillin prescriptions during the final two study years, although the impact on our findings is uncertain. *In vitro* studies have noted the emergence of daptomycin non-susceptibility in MSSA isogenic mutants with *blaZ* deletions (13, 14). Such findings may be related to the previously noted inverse relationship between β-lactam and glycopeptide susceptibility in *S. aureus*, often referred to as the “see-saw effect” (15, 16). Sakoulas and Nizet recently reported on the temporal rise in PSSA coinciding with the licensure of daptomycin (17). Thus, it is conceivable that the use of non-β-lactam antibiotics could be indirectly selecting for PSSA. Such a relationship may be of high clinical impact, as daptomycin non-susceptibility in *S. aureus* has been associated with reduced killing by host antimicrobial peptides (18), hinting at increased virulence potential of these organisms. Notably, however, no subject in our study had previous exposure to daptomycin, and we did not observe a higher rate of daptomycin non-susceptibility in PSSA.

In multivariable analyses, penicillin susceptibility was independently associated with hospital admission as well as the need for operative intervention. The reasons for the observed associations with negative outcomes are unclear but may potentially be related to the genetic background of PSSA. Studies of bloodstream isolates from a center in Sweden suggest that PSSA may be related to clonal expansion of a small number of strain types, particularly clonal complex (CC) 5 and CC45 (19). Likewise, in our previous osteoarticular infection study, PSSA disproportionately belonged to ST1 (10). In the present work, a large proportion of PSSA SSTI were also variants of ST1, although isolates represented 12 different STs. Alternatively, the observation of a more severe clinical phenotype in PSSA raises questions regarding the potential trade-off between antimicrobial resistance and strain fitness in microbial physiology. Previous models of endocarditis have found reduced virulence of vancomycin intermediate *S. aureus* compared to isogenic susceptible strains (20), and it is possible that similar relationships exist between PSSA and PR-MSSA. The contribution of other virulence factors to disease severity in PSSA is uncertain. Previous investigations have shown that MRSA strains belonging to the USA300/ST8 genotype are associated with severe skin infections with a high rate of recurrence (21). USA300 strains frequently carry Panton-Valentine leukocidin (PVL), an exotoxin associated with large abscesses. By contrast, we observed that PSSA had comparable rates of PVL carriage as PR-MSSA. Previous work has noted that *agr*III strains are associated with a prolonged bacteremia phenotype (22) as well as a higher rate of treatment failure in MSSA osteomyelitis (23). In this skin infection study, however, the majority of PSSA belonged to *agr*I. Thus, the mechanism of enhanced disease severity in PSSA remains elusive. Further work utilizing genome-wide association studies and/or models of disease is needed to investigate the associations of PSSA and disease severity.

There are limitations to this work which should be acknowledged. Foremost, our findings are from a single center and may not be generalizable. Additionally, the retrospective design limits the degree to which PSSA can be definitively linked with clinical phenotypes. The study size was relatively small, restricting conclusions that can be drawn from these data. However, isolates were selected in a random fashion from a very large surveillance database with equal distribution across the time period, adjusting for potential seasonal variation and sampling error. Finally, our study design is unable to explain the observed increased severity of disease in PSSA infections.

In conclusion, a small but significant proportion of MSSA SSTI isolates are penicillin susceptible at a large US pediatric center. PSSA were independently associated with higher rates of hospital admission and need for surgery, suggesting the potential contribution of microbial genetic background to clinical outcomes. Further efforts should focus on evaluating the emergence and increased virulence of PSSA infections.

## SUPPLEMENTAL MATERIAL

Fig. S1 spectrum.00869-24-s0001.tif Temporal trends in the relative proportion of PSSA and PR-MSSA in study period.

Fig. S2 spectrum.00869-24-s0002.tif Outpatient penicillin and aminopenicillin prescribing 2011-2021.

Table S1 spectrum.00869-24-s0003.docx Associations with need for operative debridement with general anesthesia for MSSA SSTI.

ASM does not own the copyrights to Supplemental Material that may be linked to, or accessed through, an article. The authors have granted ASM a non-exclusive, world-wide license to publish the Supplemental Material files. Please contact the corresponding author directly for reuse.
