# Supplementary figures and images for: Penicillin susceptibility among Staphylococcus aureus skin and soft tissue infections at a children’s hospital
Source: Microbiol Spectr. 2024 Sep 9;12(10):e00869-24. doi: 10.1128/spectrum.00869-24 (PMC11448063; doi:10.1128/spectrum.00869-24)

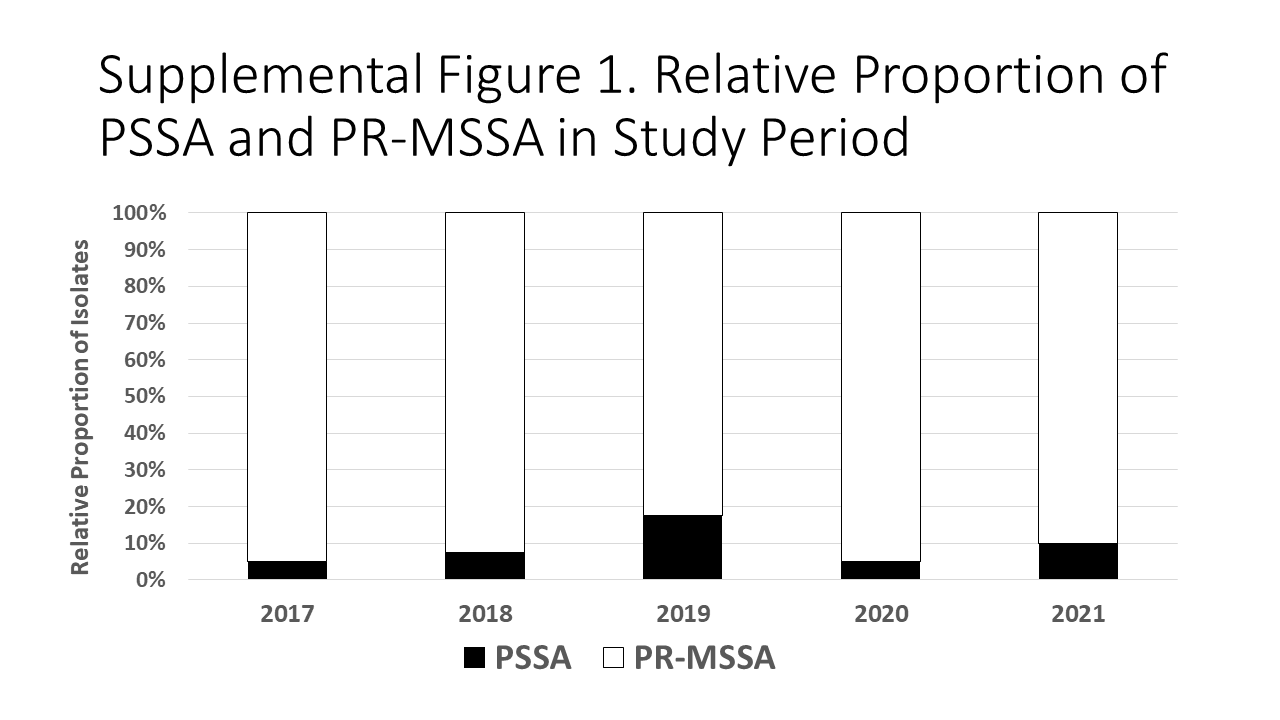

Supplement: Fig. S1 — Temporal trends in the relative proportion of PSSA and PR-MSSA in study period. [file spectrum.00869-24-s0001.tif]

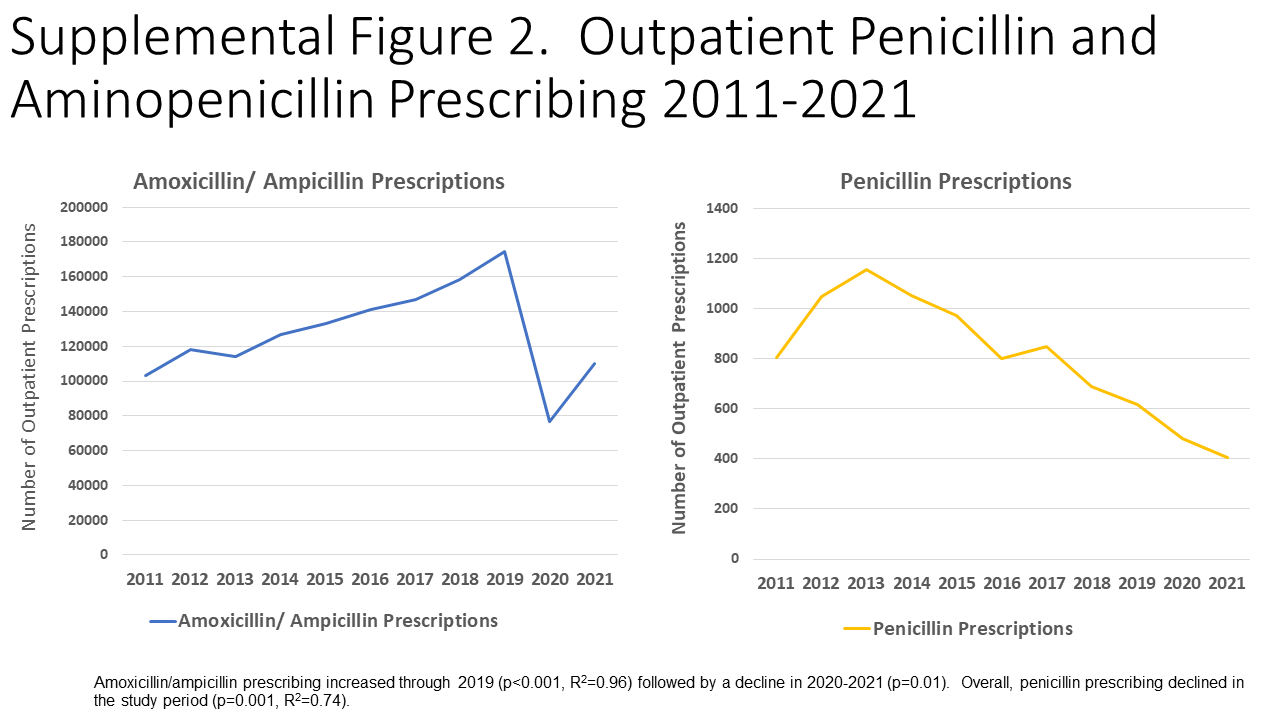

Supplement: Fig. S2 — Outpatient penicillin and aminopenicillin prescribing 2011-2021. [file spectrum.00869-24-s0002.tif]
