# Supplementary material for: Penicillin susceptibility among Staphylococcus aureus skin and soft tissue infections at a children’s hospital
Source: Microbiol Spectr. 2024 Sep 9;12(10):e00869-24. doi: 10.1128/spectrum.00869-24 (PMC11448063; doi:10.1128/spectrum.00869-24)
Supplement: Table S1 — Associations with need for operative debridement with general anesthesia for MSSA SSTI. [file spectrum.00869-24-s0003.docx]

Supplemental Table. Associations with Need for Operative Debridement with General Anesthesia

|  | Operative Debridement, n=57 | No Operative Debridement, n=143 | Univariable P | Multivariable P | aOR (95% CI) |
| --- | --- | --- | --- | --- | --- |
| Age < 4 years | 37 (64.9) | 61 (42.6) | 0.005 | 0.15 | 1.7 (0.83-3.56) |
| Infection of Neck | 14 (24.5) | 4 (2.8) | <0.001 | <0.001 | 12.41 (3.54-43.42) |
| Infection of Genitals | 10 (17.5) | 5 (3.5) | 0.002 | <0.001 | 8.78 (2.62-29.41) |
| Previous Antibiotics | 32 (56.1) | 40 (27.9) | <0.001 | 0.003 | 3.1 (1.47-6.44) |
| Previous SSTI | 8 (14) | 34 (23.8) | 0.18 |  |  |
| PVL-Positive | 21 (36.8) | 62 (43.3) | 0.43 |  |  |
| PSSA | 10 (17.5) | 8 (5.6) | 0.01 | 0.006 | 4.83 (1.57-14.79) |
